# RETRACTED: Marino et al. Aggravation of TGFβ1-Smad Pathway and Autoimmune Myocarditis by Fungicide (Tebuconazole) Exposure. *Int. J. Mol. Sci.* 2023, *24*, 11510

**DOI:** 10.3390/ijms27156778

**Published:** 2026-07-29

**Authors:** Ylenia Marino, Alessia Arangia, Ramona D’Amico, Marika Cordaro, Rosalba Siracusa, Daniela Impellizzeri, Enrico Gugliandolo, Roberta Fusco, Salvatore Cuzzocrea, Rosanna Di Paola

**Affiliations:** 1 Department of Chemical, Biological, Pharmaceutical and Environmental Sciences, University of Messina, 98166 Messina, Italy; ylenia.marino@studenti.unime.it (Y.M.); alessiaarangia@gmail.com (A.A.); rdamico@unime.it (R.D.); rsiracusa@unime.it (R.S.); dimpellizzeri@unime.it (D.I.); salvator@unime.it (S.C.); 2 Department of Biomedical, Dental and Morphological and Functional Imaging, University of Messina, Consolare Valeria, 98100 Messina, Italy; cordarom@unime.it; 3 Department of Veterinary Sciences, University of Messina, 98168 Messina, Italy; egugliandolo@unime.it (E.G.); dipaolar@unime.it (R.D.P.)

The journal retracts the article titled “Aggravation of TGFβ1-Smad Pathway and Autoimmune Myocarditis by Fungicide (Tebuconazole) Exposure” [1], cited above.

Following publication, concerns were brought to the attention of the Editorial Office regarding the integrity of Figure 3 presented in this article.

Following the journal’s standard procedures, an investigation was conducted by the Editorial Office and Editorial Board which identified duplication between panels E and F presented in Figure 3 and raised additional concerns regarding the consistency of the reported data. Although the authors cooperated during the investigation, the explanations and supporting materials provided were not deemed sufficient by the Editorial Board to resolve the identified concerns. As a result, the Editorial Board has lost confidence in the reliability of the findings, and has decided to retract this publication, as per MDPI’s retraction policy.

This retraction was approved by the Editor-in-Chief of the *International Journal of Molecular Sciences*.

The authors have been informed of this retraction but did not provide a comment on this decision.
